# Identification and characterization of methylation-mediated transcriptional dysregulation dictate methylation roles in preeclampsia

**DOI:** 10.1186/s40246-020-0256-9

**Published:** 2020-01-30

**Authors:** Shuyu Zhao, Nan Lv, Yan Li, Tianyi Liu, Yuhong Sun, Xiaodan Chu

**Affiliations:** Third Ward of Obstetrics and Gynecology, The Second Affiliated Hospital of Harbin Medical University, 246 XueFu Road, Harbin, 150006 Heilongjiang People’s Republic of China

**Keywords:** Preeclampsia, Methylation, Transcriptional dysregulation, Drug repurposing, Biomarker

## Abstract

**Background:**

Preeclampsia (PE) is a heterogeneous, hypertensive disorder of pregnancy, with no robust biomarkers or effective treatments. PE increases the risk of poor outcomes for both the mother and the baby. Methylation-mediated transcriptional dysregulation motifs (methTDMs) could contribute the PE development. However, precise functional roles of methTDMs in PE have not been globally described.

**Methods:**

Here, we develop a comprehensive and computational pipeline to identify PE-specific methTDMs following TF, gene, methylation expression profile, and experimentally verified TF-gene interactions.

**Results:**

The regulation patterns of methTDMs are multiple and complex in PE and contain relax inhibition, intensify inhibition, relax activation, intensify activation, reverse activation, and reverse inhibition. A core module is extracted from global methTDM network to further depict the mechanism of methTDMs in PE. The common and specific features of any two kinds of regulation pattern are also analyzed in PE. Some key methylation sites, TFs, and genes such as IL2RG are identified in PE. Functional analysis shows that methTDMs are associated with immune-, insulin-, and NK cell-related functions. Drug-related network identifies some key drug repurposing candidates such as NADH.

**Conclusion:**

Collectively, the study highlighted the effect of methylation on the transcription process in PE. MethTDMs could contribute to identify specific biomarkers and drug repurposing candidates for PE.

## Background

Preeclampsia (PE) is a hypertensive disorder during pregnancy and is a leading cause of maternal and fetal illness and mortality [1]. PE originates in the placenta, and the main clinical symptoms are hypertension and proteinuria in pregnant women [2]. Approximately 10~15% maternal deaths are related with PE and eclampsia [3]. Women still take an increased risk of cardiovascular events in later life, even after pregnancy subsides in PE [4, 5]. The long-term risks of diabetes mellitus, kidney disease, thromboembolism, hypothyroidism, and impaired memory are also increased [6].

Furthermore, risks containing preterm birth and all corresponding conditions, neonatal thrombocytopenia, and restricted fetal angiogenesis for the fetus of PE pregnancy women are ratchetted up [7]. The risk factors for the occurrence and development of PE are extensively researched and mainly include history of PE, chronic hypertension, gestational diabetes mellitus, antiphospholipid syndrome, and obesity [8]. However, the clinical definition and treatment for PE changed relatively little in past 60 years. Genetic and epigenetic factors for PE have been also extensively studied [9, 10]. Lang et al. report the role of NUDT21 in microRNA-binding sites of EZH2 gene increases the risk of PE [11]. Gene VHL is a novel target of E2F4-mediated transcriptional repression in PE [12]. Than et al. revealed that the placental expression of Chr19 cluster galectins [13]. In addition, more and more studies focus on epigenetic patterns of PE. Lang et al. suggest altered DNA methylation and transcription of WNT2 and DKK1 genes in placentas are associated with early-onset PE [14]. PE is associated with hypermethylation of IGF-1 promoter mediated by DNMT1 [15]. The methylation levels are changed at differential methylated regions of MEST and DLK1 in fetus of PE [16]. All the evidences suggest the importance of studying the genetic and epigenetic factors to explore the mechanism and treatment for PE.

Gene transcription could be strictly regulated by transcription factors (TFs) through binding to genomic cis-regulatory elements in a specific sequence motif. The ability of a TF to regulate its targets is modulated by a variety of genetic and epigenetic mechanisms [17, 18]. van Dijk et al. reported that the alteration of methylation for TF STOX1 could contribute to regulate its downstream target genes in PE [19]. The methylation patterns of FN1, FOS, and ITGA5 are related with TF networks in PE [20]. Lots of evidence suggest that methylation could be important modulators for the process of TFs which regulate their target genes. However, the studies focused on methylation-mediated changes in TF activity in PE are lacking.

In the present study, an integrated and calculated method is developed to identify methylation-mediated transcriptional dysregulation motifs (methTDMs) based on TFs, genes and methylation expression profiles, and experimentally verified interactions in PE. A global methTDM network is constructed, and topological features are analyzed in PE. Six kinds of patterns including relax inhibition, intensify inhibition, relax activation, intensify activation, reverse activation, and reverse inhibition patterns for methTDMs are defined, and they show specific characteristics in PE. A core module is identified to dissect roles of methylation sites in PE. The diverse and common characteristics for the six patterns are also analyzed. Functional analysis shows the methTDMs are significantly associated with metabolic-related GO terms, cytokine-cytokine receptor interaction pathway, and immune-related pathways. In addition, a drug-related network is constructed based on methTDMs to identify drug repurposing candidates for PE. Overall, the present study accumulates the understanding about the functions and mechanisms of methylation and provides novel insights, overcoming the treatment of PE.

## Results

### Inferring PE-specific methTDMs and constructing a global methTDM network for PE

**Fig. 1:**
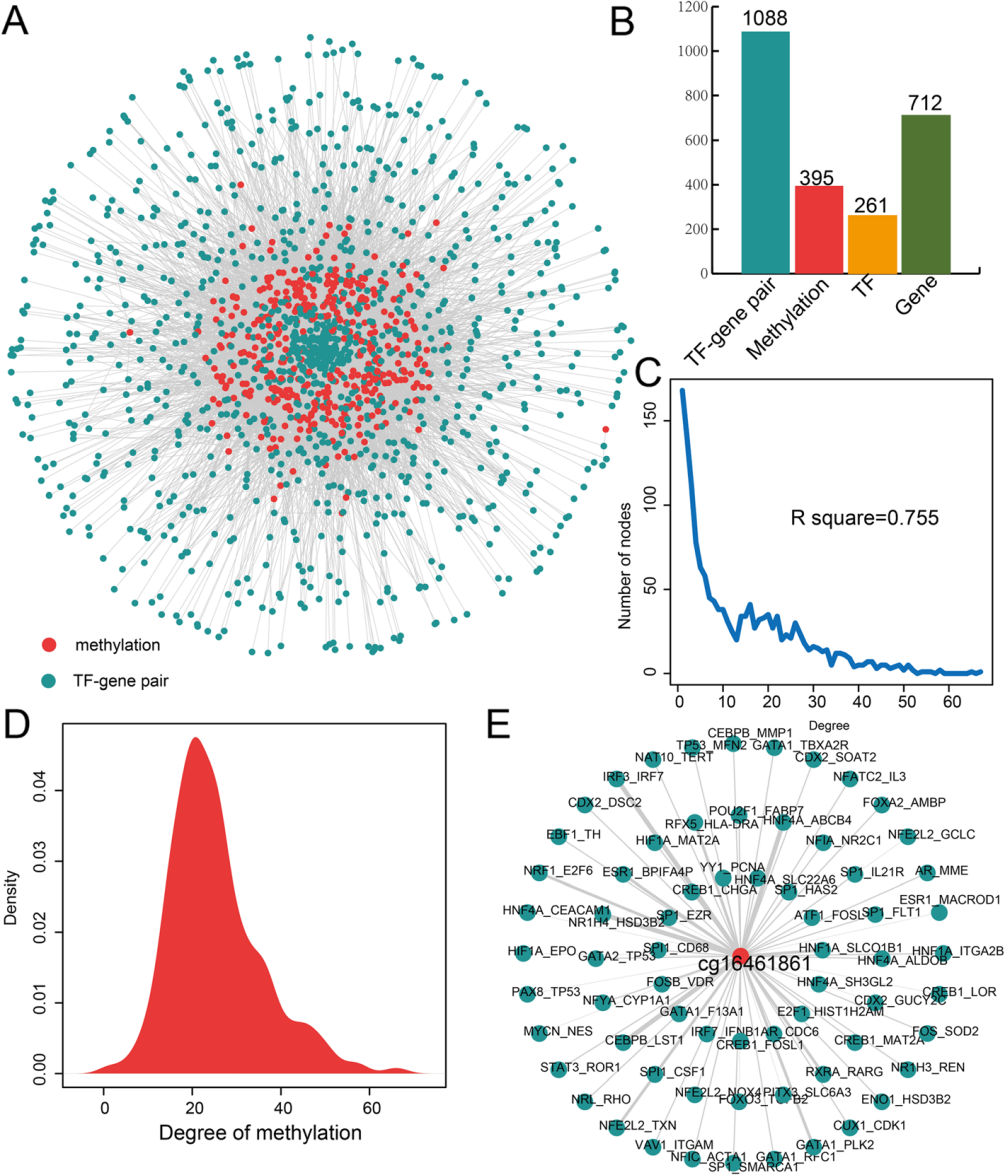
Identification and construction of methTDM network for PE. **a** methTDm network for PE. The blue and red nodes represent TF-gene pairs and methylations. **b** The bar plot shows the number of TF-gene pairs, methylations, TFs, and genes. **c** The plots show the degree distribution for PE. **d** The density distribution curve shows the distribution for degree of methylation in the global methTDM network in PE. **e** The cg16461861-mediated sub-network extracted from the global methTDM network. The thickness of lines represents the change level for transcriptional process

An integrated and calculated approach is performed, and 10,012 PE-specific methTDMs are identified. A global methTDM network is constructed, and this network contains 1483 nodes and 10,012 edges (Fig. 1a). There are 1088 TF-gene pairs, 395 methylation sites, 261 TFs, and 712 genes in this network (Fig. 1b). The degrees of all nodes in this network show scale-free distribution, and this kind of distribution indicates that the network is a typical biological regulatory network (*R* square = 0.755, Fig. 1c). We discovered that most of methylation sites influence multiple TF-gene interaction pairs. Thus, we further analyze the degree of all the methylation sites in this network and find the degrees of most methylations are centralized between 20 and 30 (Fig. 1d). This result indicates that connection of this network is close and methylations play important roles in this regulatory network. The methylation with highest degree is cg16461861 which could influence the interaction action of 67 TF-gene interaction pairs. A cg16461861-related sub-network is extracted from the global methTDM network (Fig. 1e). The cg16461861 is located at gene AHRR. Many previous studies reported that the changes of methylation sites in AHRR are associated with cancer, lung function, and low birth weight [21–23]. Although cg16461861 influence multiple TF-gene interactions, the influence levels are diverse. For example, CEBPB_LST1 pair has more positive correlation in samples with low methylation level than samples with high methylation level. However, HNF4A_ABCB4 pair was negatively correlated in samples with high methylation level and positively correlated in samples with low methylation level. All above results show that methylation is essential in the process of TF regulate gene and the mechanism is complex.

### PE-specific methTDMs are multiple and complex in PE

**Fig. 2:**
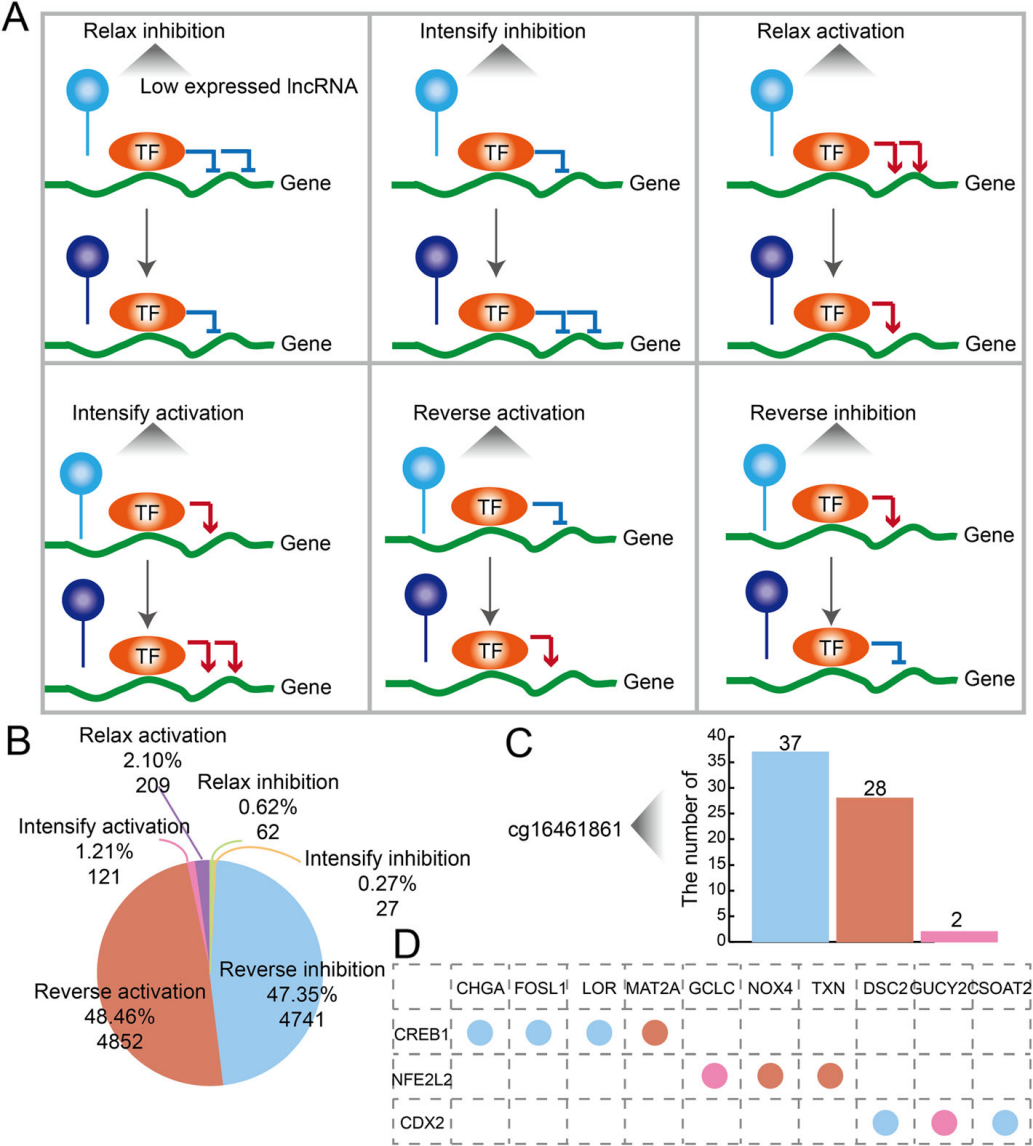
The multiple and complex regulation patterns of methTDMs in PE. **a** Six kinds of regulation patterns of methTDMs in PE. **b** The pie chart shows the distribution of regulation patterns in PE. Orange, blue, yellow, green, purple, and pink represent reverse activation, reverse inhibition, intensify inhibition, relax inhibition, relax activation, and intensify activation, respectively. **c** The bar plot shows the number of diverse regulation patterns for cg16464861 in PE. **d** The regulation patterns of cg16461861-mediated methTDMs in PE

For all we know, TFs could activate or inhibit gene expression to play their roles in biological process and the change of regulation relationship would contribute to many kinds of diseases. Besides, methylation levels would affect the regulation action of TFs on their target genes. We define this kind of methylation-mediated TF-gene interactions as methTDMs. The patterns of methTDMs in PE are multiple and complex. In order to explore in more detail the mechanism of methTDMs in PE, we classify all the PE-specific methTDMs to six kinds of patterns follow the regulatory direction of methylations and TFs (Fig. 2a). In total, there are respectively 2.10%, 1.21%, and 48.46% of methTDMs which are methylation relaxed, intensified, and reversed in the TF-gene activation regulations. There are respectively 0.62%, 0.27%, and 47.35% of methTDMs which are methylation relaxed, intensified, and reversed in the TF-gene inhibition regulations (Fig. 2b). Most methTDMs are reverse patterns, and the result indicates that methylations have significant impact on TF-gene regulations. We further analyze the regulation patterns of methylation cg16461861. The methTDMs mediated by cg16461861 have three regulation patterns including intensify inhibition, reverse activation, and intensify activation (Fig. 2c). Major TFs in these cg16461861-mediated methTDMs are CREB1, NFE2L2, and CDX2 (Fig. 2d). The same TF could regulate many genes, and these processes are also could be influenced by the same methylation following diverse regulation patterns. The result indicates that the regulation patterns of methTDMs are multiple and complex in PE.

### Some core modules are identified and show closer connections in PE

**Fig. 3:**
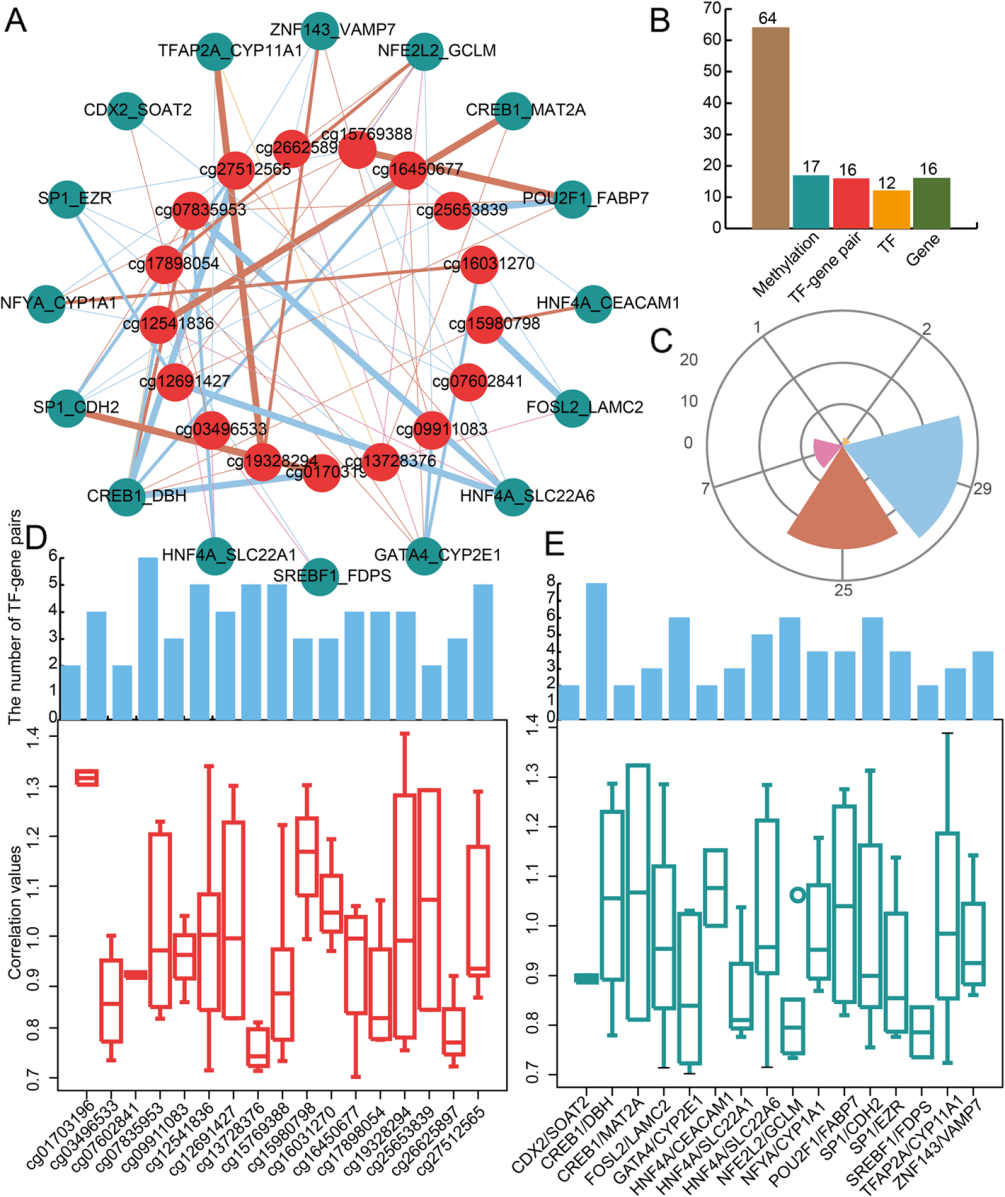
**a** The core module extracted from the global methTDM network in PE. The color of edges represents the different regulation patterns in PE. **b** The bar plot shows the number of methTDMs, TF-gene pairs, methylations, TFs, and genes. **c** The wind direction rose map shows the number of regulation patterns of core module in PE. **d** The bar plots show the number of TF-gene pairs for each methylation in core module. The box plots show the change level of TF-gene regulation for each methylation in core module. **e** The bar plots show the number of methylations for each TF-gene pairs in core module. The box plots show the change level of TF-gene regulation for each TF-gene pair in core module

A core module is extracted from the global methTDM network, and this core module contains 33 nodes and 64 edges based on module scores (Fig. 3a). The connections of this core module are more dense compared to the global methTDM network and indicate that the core module may play an important role in PE. This core module contains 64 methTDMs, 17 methylation sites, 16 TF-gene pairs, 12 TFs, and 16 genes (Fig. 3b). The core module has four kinds of regulation patterns including reverse activation, reverse inhibition, intensify activation, and intensify inhibition (Fig. 3c). Moreover, all the methylation sites are analyzed. All the changes of correlation values which are affected by methylation are obvious (Fig. 3d). Most methylation sites regulate three or more TF-gene pairs in PE. For example, methylation site cg01703196 regulates two TF-gene pairs including SP1_CDH2 and CREB1_DBH. The changes of correlation values are 1.31 and 1.29, respectively. The same TF-gene pair is also regulated by multiple methylation sites (Fig. 3e). For example, CREB1 and DBH interaction pairs are regulated by eight methylation sites including cg01703196, cg07835953, cg12541836, and cg17898054. The result indicates that methylation sites and TF-gene pairs show close connections in the core module for PE.

### Common and specific features of multiple patterns of methTDMs in PE

**Fig. 4:**
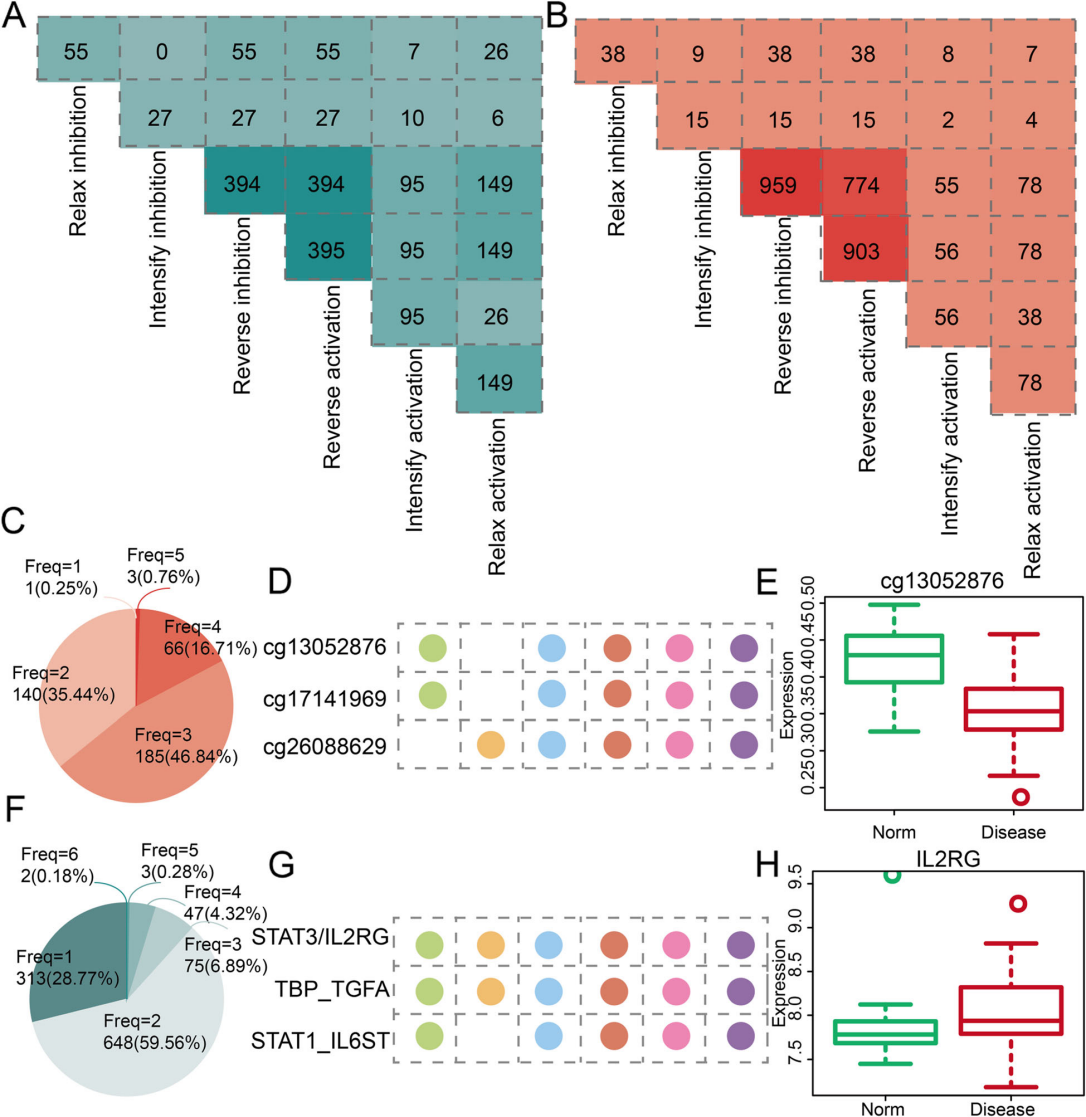
The common and specific features between any two kinds of regulation patterns based on methTDMs in PE. **a** The common TF-gene pairs between any two kinds of regulation patterns based on methTDMs in PE. **b** The common methylations between any two kinds of regulation patterns based on methTDMs in PE. **c** The pie chart shows the distribution of frequency of methylation-mediated regulation patterns in PE. **d** The regulation patterns of methylation cg13052876, cg17141969, and cg26088629. **e** The box plot shows the expression level of methylation cg13052876. **f** The pie chart shows the distribution of frequency of TF-gene pair-mediated regulation patterns in PE. **g** The regulation patterns of TF-gene pairs including STAT3/IL2RG, TBP/TGFA, and STAT1_IL6ST. **h** The box plot shows the expression level of gene IL2RG

In order to depict the mechanism of methTDMs in PE, we show the common and specific features of multiple patterns. We respectively show the common methTDMs between any two regulatory patterns for TF-gene pairs and methylations (Fig. 4a, b). The difference of intersection between two regulatory patterns is diverse. For example, the TF-gene pairs (149) in relax activation all participate in reverse inhibition and reverse activation. However, there are only six common TF-gene pairs between intensify inhibition and relax activation. For methylation sties, there are 774 common methylation sites between reverse inhibition and reverse activation. Similar to TF-gene pair, there are only four common methylation sites between intensify inhibition and relax activation. The frequency of methylation sites and TF-gene pairs for diverse regulation patterns is also analyzed. 64.56% methylation sites would participate in more than two kinds of regulatory patterns (Fig. 4c). There are three methylation sites including cg13052876, cg17141969, and cg26088629 participating in five kinds of regulatory patterns (Fig. 4d). Moreover, the methylation level of cg13052876 is differential between norm and PE samples (Fig. 4e). For TF-gene pairs, there are 40.44% TF-gene pairs participating in more than two kinds of regulatory patterns (Fig. 4f). The differences in the expression of other genes and methylations are shown in Additional file 1: Figure S1. There are two TF-gene pairs including STAT3_IL2RG and TBP_TGFA participating in six kinds of regulatory patterns (Fig. 4g). Gene IL2RG is also differentially expressed between norm and PE samples (Fig. 4h). All the results indicate that some methylation sites and TF-gene pairs play more important roles in methTDMs for PE.

### Functional analysis for TFs and genes and drug repurposing candidates for PE based on PE-specific methTDMs

**Fig. 5:**
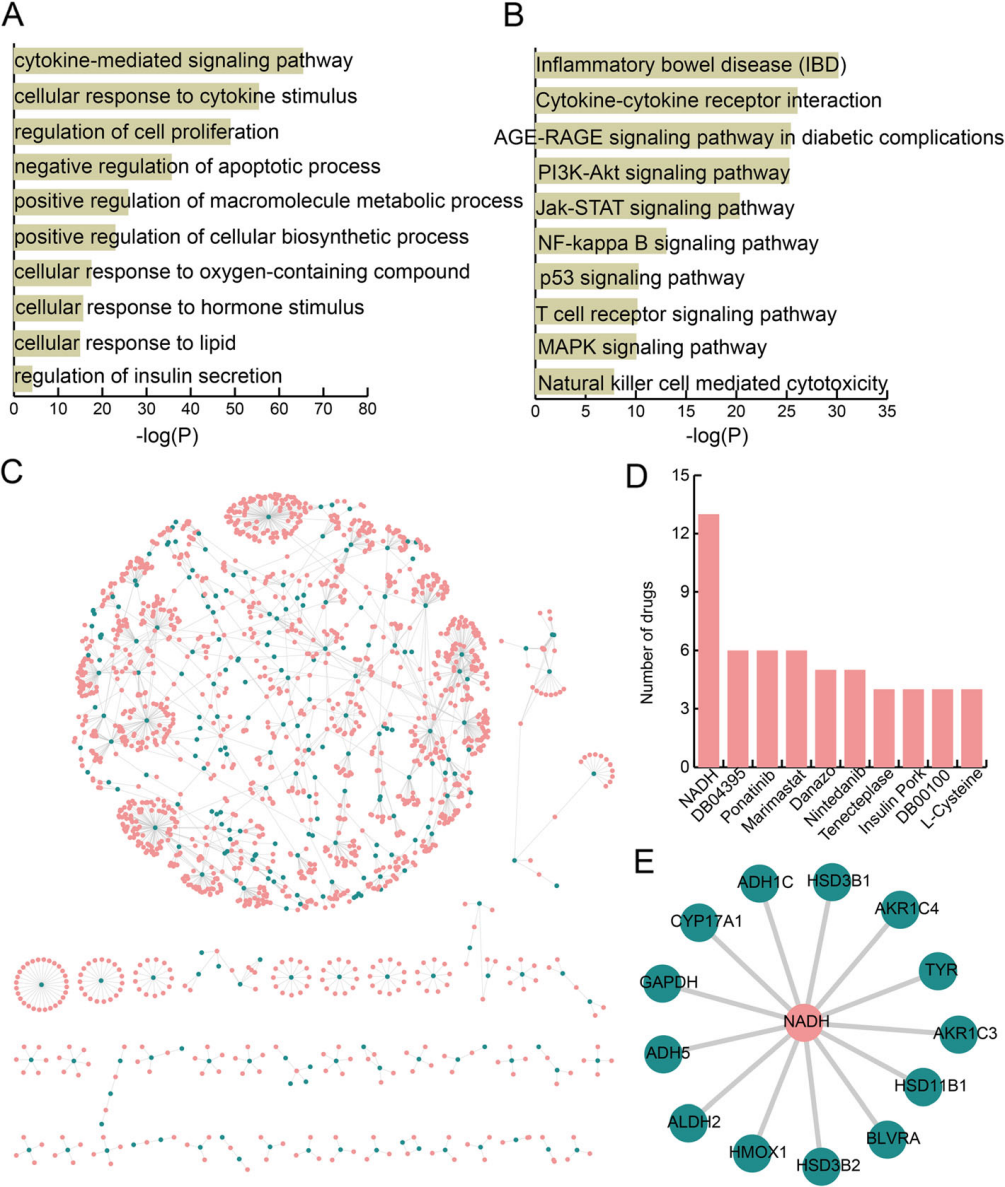
Functional analysis and drug repurposing for methTDMs in PE. **a** GO terms enriched for genes and TFs in methTDMs for PE ranked by −log10(*P*) are presented as bar plots. **b** KEGG pathways enriched for genes and TFs in methTDMs for PE ranked by −log10(*P*) are presented as bar plots. **c** Drug-related network based on methTDMs in PE. Pink and blue represent drugs and target genes in methTDMs. **d** The bar plot shows the degree of top ten drugs. **e** A drug NADH-related sub-network in PE

We perform functional analysis about KEGG pathways and GO terms for TFs and genes in PE-specific methTDMs. Functional analysis is helpful to characterize the functions of methTDMs and explore the mechanism of PE. We discover that the TFs and genes in PE-specific methTDMs are enriched in some critical GO terms such as cytokine-mediated signaling pathway, cellular response to cytokine stimulus, regulation of cell proliferation, and negative regulation of apoptotic process (Fig. 5a). Many previous studies have suggested that circulating inflammatory cytokines are implicated in the pathogenesis of PE [24–27]. These TFs and genes are also enriched in cellular response to hormone stimulus. Circulating hormone in maternal plasma is related with gestational age and severity of PE [28]. Regulation of insulin secretion is also a key enrichment GO term, and midtrimester maternal insulin resistance is associated with subsequent PE [29]. In addition, some key pathways such as cytokine-cytokin receptor interaction, AGE-RAGE signaling pathway in diabetic complications, and T cell signaling pathway are also discovered (Fig. 5b). Natural killer (NK) cell-mediated cytotoxicity is also a key enrichment pathway, and NK cells are thought to play an important role in normal placental development, have been noted recently to induce angiogenic factors and vascular remodeling, and play essential roles in PE [30]. In order to identify drug repurposing candidates for PE, a drug-related network is constructed based on PE-specific methTDMs (Fig. 5c). The network contains 1695 drugs and 308 genes. We discover 10 drugs with higher degree in this network (Fig. 5d). Specially, drug NADH shows the highest degree and some evidence suggests that NADH might be useful in treating Parkinson’s disease, chronic fatigue syndrome, Alzheimer’s disease, and cardiovascular disease [31]. In our analysis, NADH has 13 target genes in PE (Fig. 5e). Matsubara and Sato revealed that NADH oxidase activity was the same with fetal growth restriction placentas [32]. NADH maybe is a potential drug for PE treatment.

## Discussion

In our study, we develop an integrated and computational pipeline to identify PE-specific methTDMs in PE. We explore how methylation regulates the process of action of TF on gene. Several recent studies have portrayed DNA methylation as a new player in the recruitment of TF within chromatin, highlighting a need to connect TF binding sites with their respective DNA methylation profiles [33]. Methylation adjacent to negatively regulating AP-1 site reactivates TrkA gene expression during cancer progression [34]. Methylation of adjacent CpG sites affects Sp1/Sp3 binding and activity in the p21 (Cip1) promoter [35]. There is some limited evidence showing methylation also contributes to the TF-gene regulations in PE. Altered DNA methylation and transcription of WNT2 and DKK1 genes in placentas are associated with PE [14]. In our analysis, the pattern of methylation regulates TF-gene regulation pairs which are systematically characterized in PE.

We systematically analyzed the regulation patterns of methTDMs in PE and majorly defined six kinds of patterns including relax inhibition, intensify inhibition, relax activation, intensify activation, reverse activation, and reverse inhibition. The regulation patterns are also complex beside multiple. The complexity is also represented by the same methylation site which could regulate multiple TF-gene pairs by diverse regulation patterns. In our analysis, methylation cg16461861 regulates 67 TF-gene pairs and contains 3 kinds of regulation patterns in PE. In addition, a same TF-gene pair is also regulated by several methylation sites. There are some common and specific methTDMs between any two kinds of regulation patterns. All the results indicate that the methylation regulates TF-gene pairs by a multiple and complex mechanism.

Functional analysis shows that methTDMs are associated with immune-related GO terms and pathways such as T cell receptor signaling pathway. The most characteristic immunological finding in preeclampsia is the activation of both the innate and adaptive immune system [36]. NK cell-mediated cytotoxicity is also found in our analysis, and NK cell is a critical pregnancy mediator altered in PE [37]. Moreover, we construct a drug-related network based on methTDMs to identify drug repurposing candidates in PE. A key candidate drug NADH is discovered in PE. NADH is found widely in nature and is involved in numerous enzymatic reactions in which it serves as an electron carrier by being alternately oxidized (NAD+) and reduced (NADH). Some of the TFs and genes in the PE-specific methTDMs had been identified by other in vitro or mouse model studies (Additional file 2: Table S1). Future studies will focus on investigating an increased number PE samples to validate the accuracy and stability of the method presented in the current study. Experiments were also needed to verify the computational results in the future work. MethTDMs could be specific biomarkers and would provide assistance for studying mechanism and identifying drug candidates for PE.

## Conclusions

Overall, our approach identifies some PE-specific methTDMs and depicts multiple regulation patterns of methTDMs in PE. A core module is extracted from the global methTDM network, and it shows closer network structure. Some key methylation site could regulate multiple TF-gene pairs by diverse regulation patterns. Similarly, some TF-gene pairs are also regulated by multiple methylations by diverse regulation patterns. Functional analysis shows that methTDMs are associated with immune-, insulin-, and NK cell-related functions. Drug-related network identifies some key drug repurposing candidates such as NADH. MethTDMs are helpful to identify specific biomarker and drug repurposing candidates for PE.

## Methods

### TF, gene, and methylation expression profiles for PE

The TF, gene, and methylation expression profiles from the same sample are downloaded from the Gene Expression Omnibus (https://www.ncbi.nlm.nih.gov/geoprofiles/). We directly downloaded the processed data. The dataset includes placenta tissues of 30 PE and 18 control samples (GSE98224, GSE75010). Both the gene expression (GSE75010) and methylation array (GSE98224) data for these 48 placentas are available. The detailed information could be found in previous published studies [38, 39]. Probe ID information Affymetrix Human Gene 1.0 ST Array was downloaded from platform GPL6244 (https://www.ncbi.nlm.nih.gov/geo/query/acc.cgi?acc=GPL19184). Average values would be represented as gene expression if multiple probe IDs matched to the same gene name. To filter methylation and genes not expressed across all samples, the items with expression and methylation values of 0 in all of the samples were excluded. Any remaining expression values of 0 were set to the minimum value of all samples, and all values were log2-transformed.

### Identification of PE-specific TF-gene interactions and methylation sites

TF-gene interaction data are downloaded from TRANSFAC [40]. The TF-gene interactions are defined as PE-related TF-gene interactions if the absolute of Pearson’s correlation coefficients (PCCs) are larger than 0.25. PCCs were calculated based on expression of TFs and genes in each TF-gene pair for PE samples. Student’s *t* test is used to identify differential methylation sites for PE between 30 PE and 18 control samples. False discovery rate (FDR) is calculated, and differential methylation sites are extracted in which FDR is 0.05 smaller. After the above filter steps, 1911 PE-specific TF-gene interactions are identified and used for follow-up analysis. All analyses were performed using R 3.2.3 statistical software.

### An integrated and computational approach for identifying methTDMs in PE

We develop an integrated and computational approach to identify methTDMs in PE. First of all, for each PE-specific methylation, the PE samples are divided into two parts including top and bottom 40% of samples in terms of methylation values. Second, the alteration levels of associations between TF and gene are considered for a given methylation site by independently testing each methylation-TF-gene motif. For a given methylation site, all the PE samples are ranked based on the methylation level. Then, the PPC values between each TF and gene interaction are respectively calculated in top and bottom 40% PE samples based on above sort. A TF-gene interaction is defined as changed interaction when the absolute difference of PCC values between top and bottom 40% PE samples is larger than 0.7. Meanwhile, this methylation-TF-gene motif is considered as a candidate methTDM for PE. All the PE-specific TF-gene interactions are calculated for a given methylation site. At last, we perform 1000 random permutations for the sample labels of expression and methylation profiles. Permutated PCCs were generated and compared with real PCCs for PE-specific methTDMs. If the PCC values with permutation were smaller than real PE-specific methTDMs, these methTDMs were considered as candidate significant methTDMs. FDR is used to correct all permutation results, and FDR < 0.05 is selected as threshold value to generate significant methTDMs for PE.

### Pattern classification of methTDMs for PE patients

We classify all the methTDMs to six kinds of patterns based on regulation forms to further explore the detailed mechanisms of methTDMs of PE. The six patterns are listed below:

1. Relax inhibition—TFs inhibit the expression of gene and methylation which could weaken this inhibition action.
2. Intensify inhibition—TFs inhibit the expression of gene and methylation which could strengthen this inhibition action.
3. Relax activation—TFs activate the expression of gene and methylation which could weaken this activation action.
4. Intensify activation—TFs activate the expression of gene and methylation which could strengthen this activation action.
5. Reverse activation—TFs inhibit the expression of gene and methylation which could invert the inhibition to activation.
6. Reverse inhibition—TFs activate the expression of gene and methylation which could invert the activation to inhibition.

### Construction and analysis of methTDM network and identifying core modules for PE

The global methTDM network for PE is constructed by Cytoscape 3.0 (http://www.cytoscape.org/). The degree analysis is also performed by Cytoscape 3.0. We use package MCODE with default parameters in Cytoscape. MCODE package could identify densely connected regions for a given network based on topology features. The package MCODE also provides scores of each module, and higher scores represent more connected modules. Finally, a core module is extracted with the highest module score.

### Functional enrichment analysis for methTDMs in PE

Functional enrichment analyses are performed with Enrichr online web tool with default parameters for all the TFs and genes in methTDMs of PE [41]. We obtained enriched Gene Ontology (GO) terms (FDR < 0.01) and Kyoto Encyclopedia of Genes and Genomes (KEGG) pathways (FDR < 0.05).

### Identification of drug repurposing candidates for PE based on methTDMs

The gene-drug interaction data are obtained from DrugBank which is a public database including comprehensive molecular information about drugs, their mechanisms, their interactions, and their targets [42]. Then, a drug-related methTDM network is constructed and analyzed to identify drug repurposing candidates for PE.

## Supplementary information

**Additional file 1:** The differences in the expression of other genes and methylations.

**Additional file 2: Table S1.** Experimentally verified PE-associated genes.

## Data Availability

All data generated or analyzed during this study are included in this published article.
