# Supplementary figures and images for: Identification and characterization of methylation-mediated transcriptional dysregulation dictate methylation roles in preeclampsia
Source: Hum Genomics. 2020 Jan 30;14:5. doi: 10.1186/s40246-020-0256-9 (PMC6993410; doi:10.1186/s40246-020-0256-9)

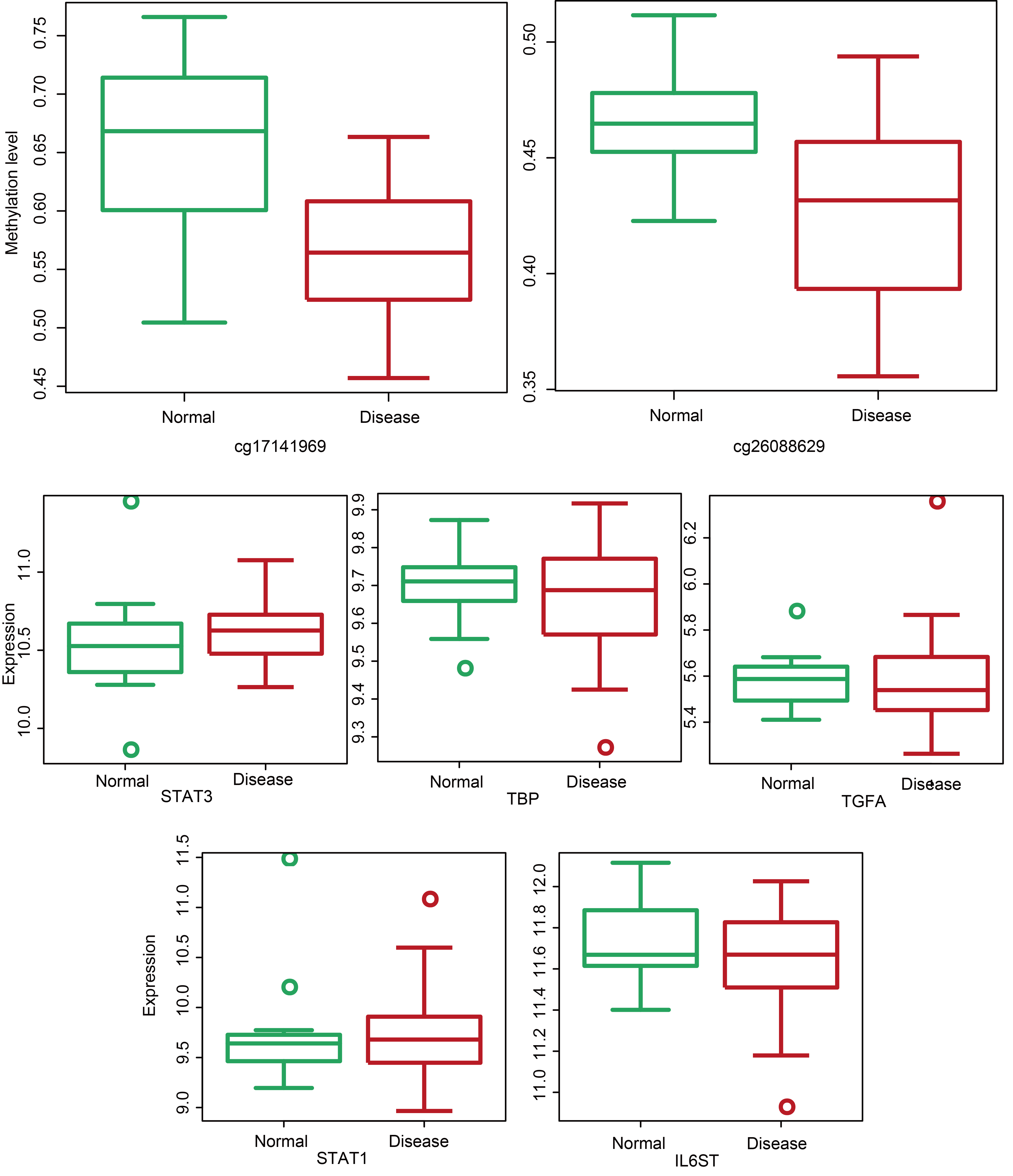

Supplement: Supplementary file 1 — Additional file 1: The differences in the expression of other genes and methylations. [file 40246_2020_256_MOESM1_ESM.tif]
